# An analysis of learner outputs in problem posing as sentence-integration in arithmetic word problems

**DOI:** 10.1186/s41039-017-0049-5

**Published:** 2017-02-10

**Authors:** Nur Hasanah, Yusuke Hayashi, Tsukasa Hirashima

**Affiliations:** Department of Information Engineering, Hiroshima University, Hiroshima, Japan

**Keywords:** Problem posing, Arithmetic word problems, Elementary school students, Learning analytics

## Abstract

Achieving practical implementation of learning by problem posing faces the issue of inefficiency due to the time needed for assessment and giving feedback to students’ posed problems. As a solution of this problem, we have developed a tablet PC-based software for learning by posing arithmetic word problems named Monsakun. The software is based on Triplet Structure Model of arithmetic word problem. In this research, we investigated problems posed by elementary school students in Monsakun to understand whether Monsakun encourages them to think about the structure of arithmetic word problems. The result shows that students did not pose problems randomly but considered things first. We also found that the frequent errors are actually meaningful errors, and students tried to pose problems satisfying as many constraints as possible, which means they actually think about the structure to pose required problems in the assignments. The process of understanding assignment requirements and relating them to suitable sentence cards is an important point especially for young learners to reach deep understanding of the structure of arithmetic word problems.

## Introduction

### Background

Two activities that have been identified to be central themes in mathematics education are problem posing and problem solving. Problem solving practice, as the most popular way of teaching the solution method, has been long integrated into school mathematics (Stanic and Kilpatrick 1988). Although not as popular, learning by problem posing has been suggested as an important way to promote learner understanding (Ellerton 1986; Polya 1957). The practice of problem posing is different than the usual practice of teaching by solving pre-formulated problems, in the way of encouraging learners to generate new problems (English 1997; Silver and Cai 1996). It is one of the important foundations of reformation in mathematics education, and the realization of its importance has led into growing research of various aspects in activities of learning by problem posing (English 1998; English 2003; National Council of Teachers of Mathematics 2000).

Arithmetic word problems remain one of the most difficult areas of teaching mathematics. Young students who have mastered simple additions and subtractions often stumbled when facing word problems which require understanding of the conceptual knowledge (Riley et al. 1983). Tasking students with the generation of a new arithmetic problem and construction of a new numerical relation can be seen as an effort toward better understanding of word problems (Brown and Walter 1990). However, achieving practical implementation of learning by problem posing faces the issue of inefficiency due to the time needed for assessment and giving feedback to students’ posed problems. While students found difficulty in posing mathematically correct problems in a satisfying amount in a given time, teachers were having problems of limited time for assessing students’ work during class activity. These problems are the main reason of the unpopularity of problem posing activity (Nakano et al. 1999).

To address this issue, several researchers have attempted to build an Intelligent Learning System to automate the problem posing assessment and incorporate the system in school practice. AnimalWatch is a web-based learning environment that enables teachers and students to create and share arithmetic word problems in fifth grade elementary school (Arroyo et al. 2001; Arroyo and Woolf 2003). This study was carried further for middle school students with the subject of arithmetic and fractions (Beal et al. 2010; Birch and Beal 2008). Another study was conducted where a learning environment systematically presented examples of problems to undergraduate students, and afterwards they are asked to build a variety of problems based on the example (Kojima and Miwa 2008; Kojima et al. 2010). Hirashima et al. (2008) targeted elementary school students in their research using an interactive problem posing learning environment named Monsakun.

The effectiveness of problem posing method has been investigated for a variety range of learners. Most research on problem posing was conducted on higher grade of school, as we have seen in undergraduate students by Kojima and Miwa (2008) and in high school students (Van Harpen and Sriraman 2013). Furthermore, research findings on middle school students were also reported in several papers (Birch and Beal 2008; Silver and Cai 1996; Walkington and Bernacki 2015). For elementary school students, the AnimalWatch by Arroyo and Woolf (2003) targeted fifth grade students, and Monsakun by Hirashima et al. (2008) was used by second grade students.

Researches of problem posing environments as mentioned above generally reported effectiveness of the problem posing practice using evaluation method of pretest and posttest comparisons. It is necessary to further analyze the learner products using the data collected by the system to get better view of learner’s problem posing process in order to capture learner’s understanding of math and science concepts (Birch and Beal 2008). The aim of this study is to investigate the learner products in problem posing, that is, posed problems. We argue that problem posing is an activity that promotes learners to think structurally about arithmetic word problems. By analysis of the products we evaluate that “learners have thought about the structure of problems” and “learners’ thinking about the structure has been improved in accordance with the progress of exercise.”

This study analyzes the posed problems on an interactive problem posing learning environment named Monsakun. Monsakun (means “Problem-posing Boy” in Japanese) is a computer-based learning environment to realize learning by problem-posing in a practical way for one operation of addition and subtraction. The software delivers the process of assessment and giving feedback to students’ posed problems automatically, enabling teachers to monitor students’ progress individually as well as all students in a classroom in a real time (Hirashima et al. 2007; Kurayama and Hirashima 2010). The development of this system was started by Nakano et al. (1999) who proposed a sentence template method for arithmetic word problem, followed by the problem template method (Hirashima et al. 2000; Nakano et al. 2002), and leading to the sentence card method (Hirashima et al. 2006), which was implemented in Monsakun.

### Purpose

Even though students seem to be highly engrossed in learning activities using computer or tablet, Dynarski et al. (2007) shows not much evidence of the software influence on higher performance of math and reading in the students. Conducting pre- and posttest is the most common way to evaluate a learning environment as seen in Beal et al. (2010), Chang et al. (2012), and Oliveira Chaves et al. (2015)). Another way is to conduct a deep analysis of the students’ behavior as seen in Biswas et al. (2005; 2010). The effectiveness of Monsakun in practical use has been reported in previous studies using pre- and posttest evaluation (Yamamoto et al. 2012) as well as investigation of university students’ thinking process when using this software (Hasanah et al. 2014; Hasanah et al. 2015). In this paper, we report on our analysis of posed problems by elementary school students on Monsakun in terms of whether Monsakun encourages learners to think about the structure of arithmetic word problems.

The purpose of Monsakun as a problem posing learning environment is to encourage students to not only pose problems but also to understand their structural nature. Monsakun provides learners with a novel way to promote learning by problem posing, and it has different aspects from other practice of problem posing activity. Through previous researches, the usefulness of Monsakun has been confirmed for learning by problem posing. This paper discusses the validity of problem posing as sentence integration in terms of learners’ activity, because problem posing task in Monsakun is conducted by making a combination of given sentences, which at first glance seems not to require deep thinking.

There are two main points to be discussed in this paper: one is whether learners pose the required problems by chance, and the other is how learners can get to the correct answer if they do not get to it by chance. This study tests the randomness of learners’ answers in Monsakun and analyzes the trend of them, especially, whether they focus on the structure of arithmetic word problems.

First, in Monsakun, the process of posing a problem is conducted by the combination of given sentences. Thus, theoretically, it is possible for learners to pose problems in random way and they can also get to correct answers stochastically, which means that they might not consider anything when posing problems. On the other hand, our aim in developing this system is to promote students’ logical ability and thinking through posing problems instead of only solving problems. Therefore, we conducted this study to investigate that students do not pose problems in a random way but with some consideration.

Second, Monsakun is based on a model called “Triplet Structure Model,” which describes the structure of arithmetic word problems (Hirashima et al. 2014). This model defines the components of arithmetic word problems and the necessary conditions of simple arithmetic word problems. These conditions also become the constraints learners must satisfy in problem posing. This study investigates the trends of posed problems with the constraint, that is, how many constraints are satisfied in them in practical uses of Monsakun.

Based on this purpose, we defined the research questions as follows: (1) Do students pose problems randomly, in relation to the natural possibility of each assignment? (2) In what way the trends of posed problems by learners could be explained with the Triplet Structure Model? This study is limited only to the type of arithmetic word problems used in Monsakun. The emphasis of analysis of students’ problem posing related to Triplet Structure Model distinguishes this study from the other problem posing research.

The composition of this paper is as follows. The “Theoretical background” section gives an overview of the structure of arithmetic word problem: the Triplet Structure Model, the types of story in simple addition/subtraction word problems, and the types of constraint based on the task model of problem posing; followed by the introduction of Monsakun. The “Methods” section explains the experiment subjects and the data analysis framework. The “Results and discussion” section discusses the result analysis of the posed problems in accordance to our research purpose. Finally, the “Conclusion” section concludes this paper and shows some prospects for future study.

## Theoretical background

It is necessary to define the structure of arithmetic word problems as the target of analysis in this study. Triplet Structure Model defines the structure with the constraints to form word problems. This section explains the model and the constraints defined in it as the basis of the analysis of posed problems as the product of problem posing. The interface of Monsakun and its practical uses also explained in this section.

### Triplet Structure Model

**Fig. 1:**
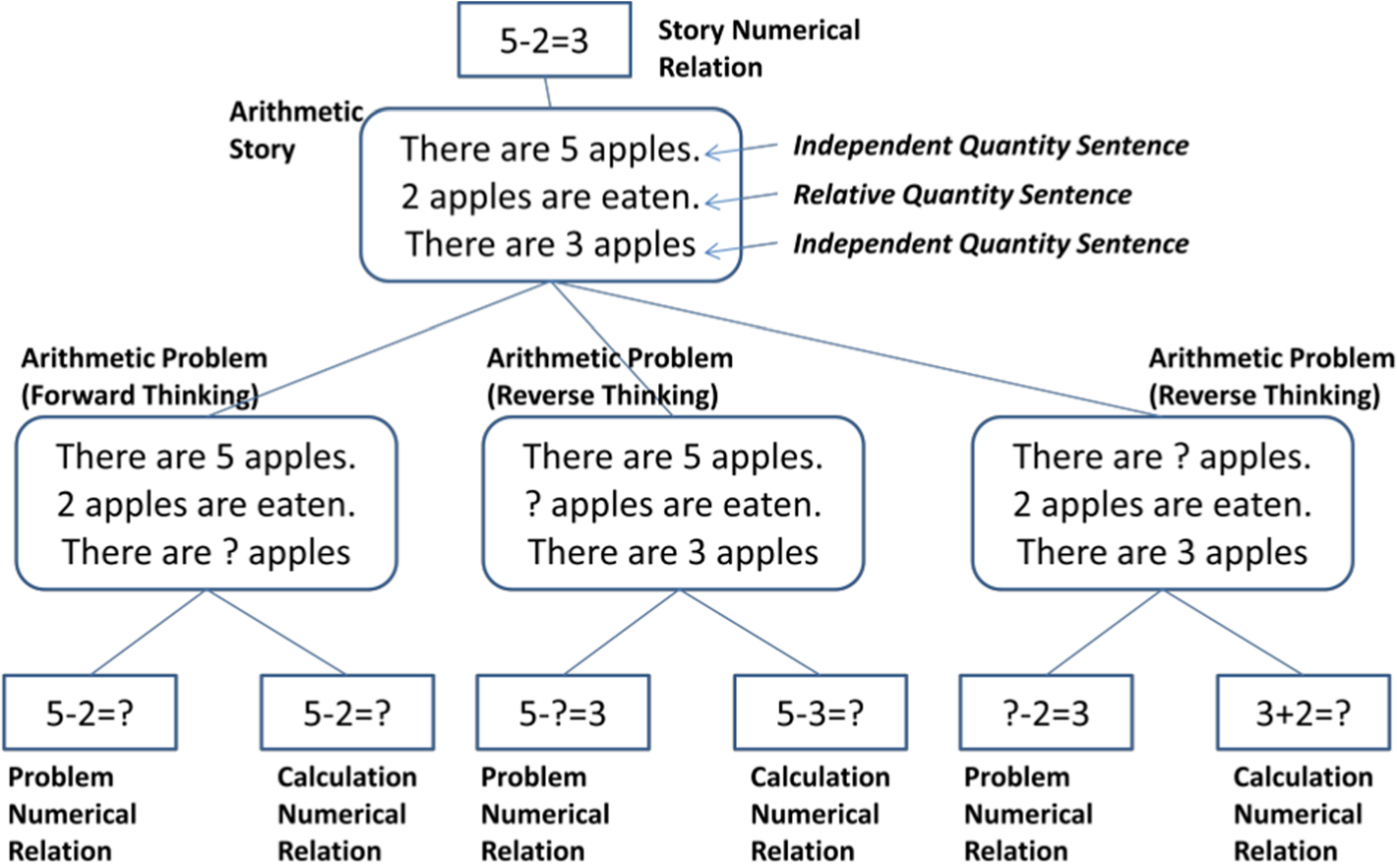
Triplet Structure Model (Hirashima et al. 2014)

Triplet Structure Model, as shown in Fig. 1, describes the components of arithmetic word problems and the basic structure of them. In this model, an arithmetic word problem is defined that it consists of three sentences including different quantities and each sentence must represent only one quantity with the meaning of them in the story. The three sentences include two “independent quantity sentences” and one “relative quantity sentence.” Independent quantity sentences describe numbers of objects, for example, “There are 5 red apples.”, “There are 3 green apples.”, and so on. Relative quantity sentences describe the relation between the other independent quantity sentences, for example, “There are 8 apples altogether.”, “2 apples are eaten.”, and so on. The combinations of different sentences form different stories and assign different roles for each sentence (Hirashima et al. 2014).

An arithmetic word problem leads two types of numerical formula: one represents the story of the problem and the other represents the calculation to solve the problem. In the example, [There are 5 apples. 3 apples are eaten. There are some (?) apples.], the former is 5 − 3 = ? and the latter is 5 − 3 = ?. On the other hand, if the problem is [There are “?” apples. 2 apples are eaten. There are 3 apples.], the former is ? − 2 = 3 and the latter is 3 + 2 = ?. The two types of formula are different. This type of problem, where the calculation and problem numerical relation are different, is called “reverse thinking problem.” This type of problem is more difficult for students than forward thinking problems, because the student is required not only to understand the story but also to derive the calculation from the story.

### Definition of story types

As mentioned above, there are two types of sentence in arithmetic word problems: independent quantity sentence and relative quantity sentence. A relative quantity sentence contains keyword determining the type of story, for example, “…eaten”, “…in total”, “…less than…” or “…more than…”. An arithmetic word problem of binary operation is integration of two independent quantity sentences and one relative quantity sentence.

There are four story types in arithmetic word problems of addition and subtraction: (1) combination, (2) increase, (3) decrease, and (4) comparison (Riley et al. 1983). In Monsakun, the differences among them are defined as differences of integration of sentences. For example, a combination story type problem consists of the followings:

- There are seven apples (independent quantity sentence),
- There are three oranges (independent quantity sentence), and
- There are ten apples and oranges in total (relative quantity sentence: combination story type)*.*

### Types of constraints based on task model of problem posing

**Fig. 2:**
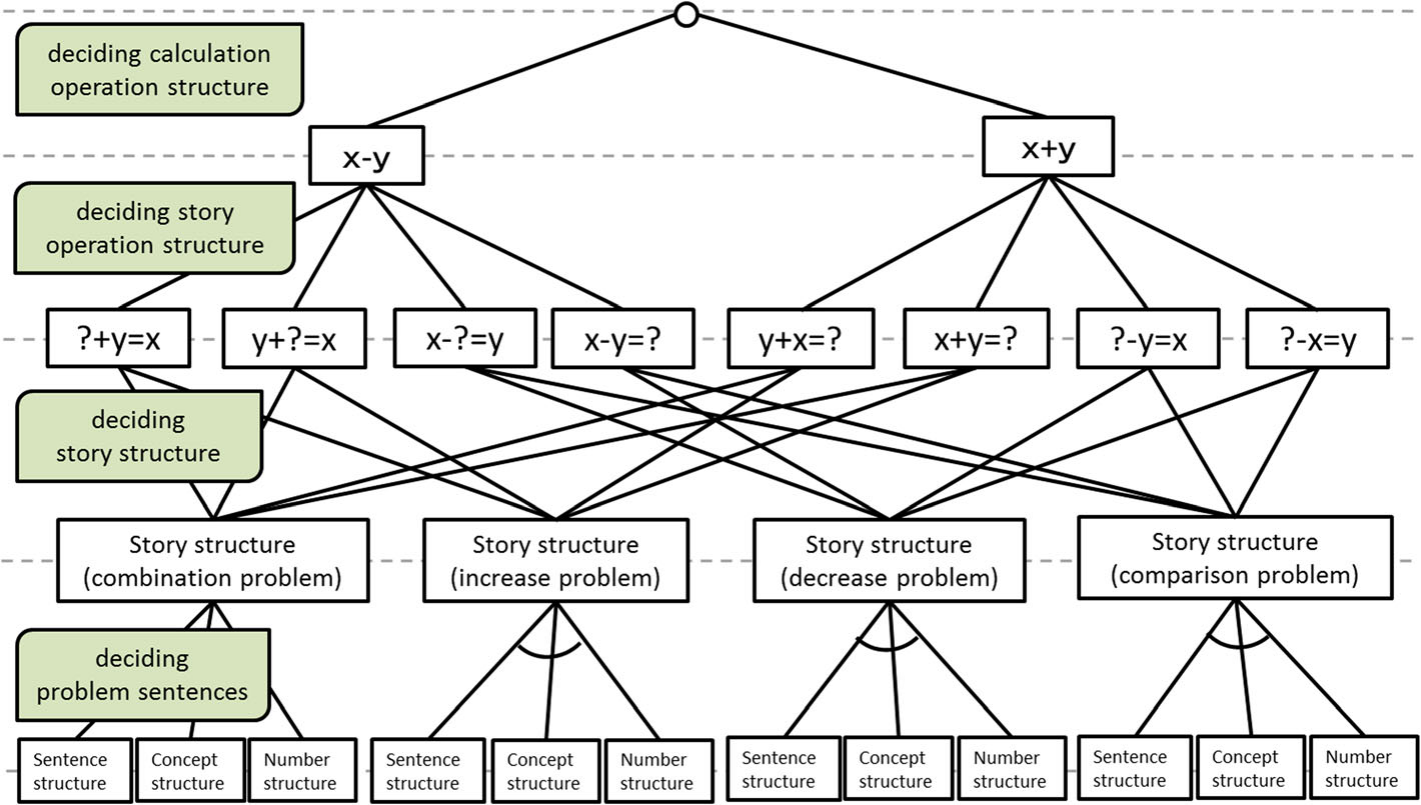
Task model of problem posing as sentence-integration

Based on the consideration of problem types in Triplet Structure Model, the task model of problem posing as sentence-integration has been developed, as shown in Fig. 2 (Kurayama and Hirashima 2010). In problem posing activity, there are four main tasks to decide: (1) calculation operation structure, (2) story operation structure, (3) story structure (story types), and (4) problem sentences. Each element has some options. Triplet Structure Model describes the essential conditions to form a problem and defines problem posing as a task to choose an option in each element from all the possible combination.

First of all, this task model illustrates all the possible valid combinations of the elements and direct and indirect relations among them. The elements are related with neighbors in order. For example, a calculation formula, “*x* + *y*” or “*x* − *y*,” is directly related to story formulas, not to story types and problem sentences.

When one element is decided, the other elements might be restricted. For example, if story operation is decided on for one of them, possible story types are narrowed to only two. For example, if the story operation of a problem is “? + *y* = *x*,” the possible story types are only combination or addition. On the other hand, even if calculation operation is decided, the possible story types are not narrowed, that is, all the story type can be made with the calculation operation.

About problem sentences, Fig. 2 does not illustrate the options. This element includes three sub-elements: sentence structure, concept structure and number structure. *Sentence structure* is the composition of sentences. As defined in Triplet Structure Model, an arithmetic word problem must consist of two independent quantity sentences and one relative quantity sentence. The type of relative quantity sentence is related to story types. *Concept structure* requires the consistency of objects in the sentences. For example, if story type is increase or decrease, objects in three sentences must be the same. On the other hand, if story type is combination or comparison, objects in the independent quantity sentences are different and both of them are in the relative quantity sentence. *Number structure* requires the consistency of numbers in the problem. Each number in the problem must be derived from the other numbers.

**Fig. 3:**
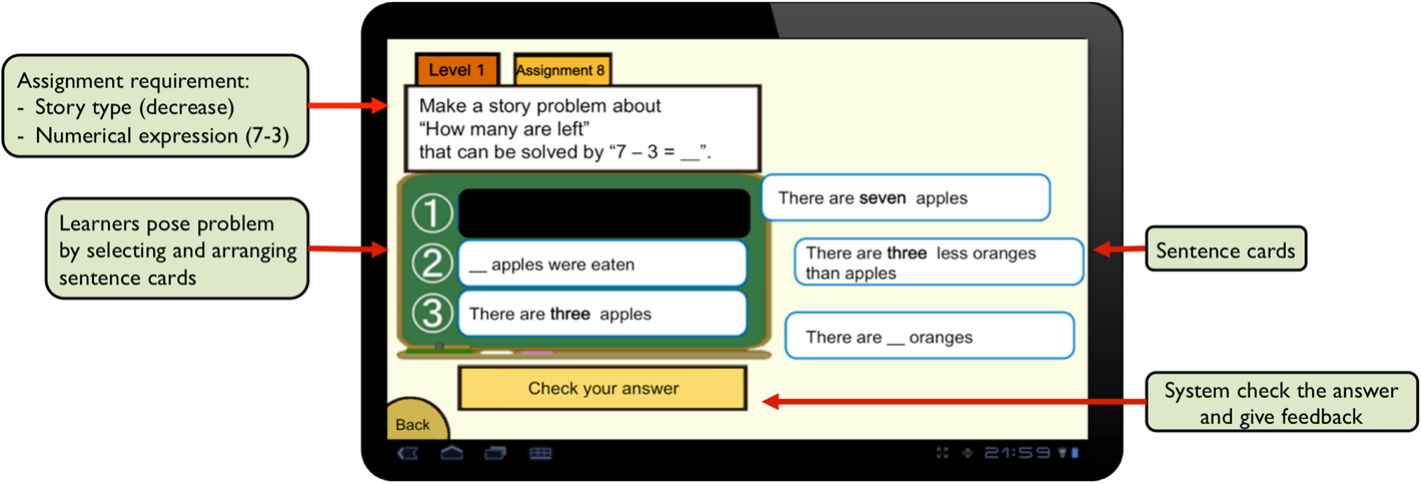
Interface of Monsakun

Problem posing is a task to an option in each element following the relations in the task model. In addition to that, assignments in Monsakun provide restrictions on formula and story type. For example, the assignment shown in Fig. 3 requires posing a problem related to the formula “7 − 3” and decrease story type. Based on the task model, what learners are required to think in this assignment is to find a combination of options in the elements that satisfy the requirement. In this case, whether a learner think the required formula is calculation formula or story formula, the learner can pose a decrease story type, because both of the calculation operation “*x* − *y*” and the story operation “*x* − *y* = ?” are related to decrease story type. On the other hand, if the requirement is the formula “7 − 3” and increase story type, it is important to identify whether the formula is calculation operation or story operation. If the required formula is story operation, it is not related to increase story type. Only when the formula is calculation operation it is related to the story type. In this case, for example, the problem {There are 3 apples/… apples were added/There are 7 apples.} satisfies the requirement.

Based on the task model of problem posing and the format of assignments in Monsakun, there are five main constraints must be satisfied in posed problems, which are the following: (1) calculation, (2) story type, (3) number, (4) concepts/objects, and (5) sentence structure. When a posed problem satisfies all five constraints, the required problem in the assignment is successfully posed. When a posed problem satisfies less than five constraints, the posed problem partially fills the requirements and the unsatisfied constraints represent the cause of the inadequateness for the requirements. This also means that the posed problem is not meaningless, because it still satisfies some constraints. When a posed problem satisfies no constraint, the problem is meaningless.

### Monsakun as learning environment for problem posing

Figure 3 shows the interface of Monsakun. In each assignment, Monsakun provides learners with a requirement to form a problem and a set of sentence cards. By selecting and arranging appropriate cards, learners pose the arithmetic word problem fulfilling the requirement. In the problem posing activity, learners do not create their own problem statements; however, they are required to interpret the sentence cards and integrate them into one problem. This activity is called “problem posing as sentence-integration” (Hirashima and Kurayama 2011). Monsakun adopts the analysis of semantic structures in arithmetic word problems by Riley et al. (1983) and the process model of problem solving of the word problems by Kintsch and Greeno (1985). Its problem posing assignments encourage learners to distinguish the extraneous information in word problems, which is more difficult than solving a standard word problem, as stated by Muth (1992).

Monsakun has five levels of assignments (the sixth level is random) which are categorized by (1) type of problem: forward or reverse thinking, (2) provided formula: story or calculation formula and (3) story types: combination, increase, decrease and comparison. Table 1 shows the setting of assignments at each level.

**Table 1:** Detailed level assignments in Monsakun

Level	Number of assignments	Type of problem thinking	Provided formula	Story types
1	12	Forward	Story	Combination, increase, decrease, comparison
2	3	Forward	Story	Increase + combination
3	12	Reverse	Story	Combination, increase, decrease, comparison
4	3	Reverse	Story	Increase + combination
5	12	Reverse	Calculation	Combination, increase, decrease, comparison
6	12	Random		

The practical use of Monsakun has been conducted in several elementary schools (Hirashima et al. 2008; Yamamoto et al. 2012; Yamamoto et al. 2013). The effect of learning by problem posing with Monsakun was investigated by the analysis of pre-test and post-test of high-score group and low-score group of the students. As a result, it has been confirmed that problem posing exercise using Monsakun is effective to improve both problem posing and problem categorization abilities. Furthermore, after long term use of Monsakun in an elementary school, the result showed that both the students and teachers enjoyed using this system continuously and considered it useful for learning.

## Methods

### Experiment subjects

To conduct the analysis, we examine the log data of Monsakun practical use from 39 first grade students in a Japanese elementary school. The practical use, as described in Yamamoto et al. (2012), was conducted in nine class sessions and Monsakun was used in seven class sessions of them, where each session starts by Monsakun use for 5 min, usual classroom teaching activity for 35 min, and concluded by Monsakun use for 5 min. The teacher was involved in every session. The teacher monitored students’ progress in real time using Monsakun Analyzer and gave assistance to students who seem to have difficulties in progressing with problem posing task in Monsakun. During the teaching activity, the teacher provided one assignment to all students with the same form of problem posing in Monsakun and let them challenged the assignment together through active discussion by all students.

During seven class sessions, students practiced all the levels in Monsakun. In Monsakun, learners try assignments step by step from the first one. A learner can move on to the next assignment when he or she gets successful in the provided one. He or she must continue to try the same assignment until getting successful in the required problem posing. In each class session students try only one level. If a learner finishes all the assignment in the level, he or she repeats the same level.

### Data analysis framework

This study investigates whether Monsakun encourages learners to think about the structure of arithmetic word problems. Using the viewpoint of Triplet Structure Model, we analyze the satisfied constraints of posed problems. If learners pose problems randomly without thinking, they would pose many meaningless problems or less meaningful problems in terms of the constraints. That is to say, they do not think about the structure of arithmetic word problems.

Firstly, the rate of finished students and the average of steps and mistakes in each level is reported to show students’ performance in posing problems with Monsakun. Then, we analyze the proportion of the numbers of satisfied constraints in actual students’ answer and possible assignment setting using chi-square test. If learners pose problems randomly, the proportion would be close to the proportion in the assignment setting. Afterwards, we analyze the difference of the proportions among assignments in the same story type. If learners pose problems with some consideration, the proportion would reflect their thoughts.

We analyze students’ log data in assignments at levels 1, 3, and 5 to find out students’ performance. We do not include levels 2 and 4 in the analysis, because they only consist of three assignments in each, and do not include assignments of all the story type.

## Results and discussion

### Comparison of students’ performance among the levels

**Fig. 4:**
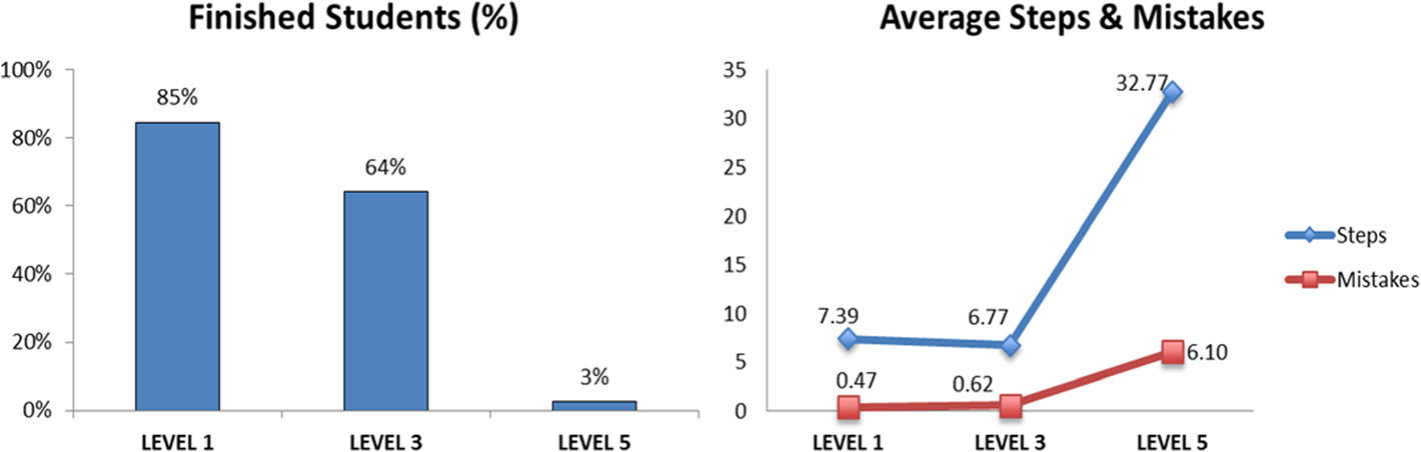
Comparison of students’ performance in levels 1, 3, and 5

The rate of finished students and the average of steps and mistakes in each level are shown in Fig. 4. Counting the first time students posed problem in each level, 85% of students were able to pose all assignments in level 1 correctly, and 64% finished level 3. In contrast, the number of students who finished all assignments in level 5 decreased very rapidly compared to levels 1 and 3.

The average of steps and mistakes shows how many steps a student needed in order to pose a correct problem in one assignment, and how many mistakes he made during the process. Ideally, a student would only need 3 steps to pose a correct problem, because a problem in Monsakun consists of the arrangement of 3 simple sentence cards. As shown in Fig. 4, the average of steps in level 3 was slightly lower than level 1, even though the average of mistakes was slightly higher, which suggests that students learned to select cards more effectively by learning from their mistakes. However, the average in level 5 was very high compared to levels 1 and 3, which shows that level 5 was indeed very challenging for students.

### Analysis of posed problems in level 5

From the analysis in the previous section, students seem to struggle hard when they are given reverse thinking problems with provided calculation formula as in level 5, in contrast of provided story formula as in levels 1 and 3. In this section, we will examine students’ posed problems in level 5.

In Monsakun, five or six sentence cards are provided in each assignment. Three of them are correct cards, which satisfy all constraints from the assignment requirement and when ordered correctly will form the correct problem. The rest are dummy cards, which designed through careful considerations by the expert as a meaningful distraction to the students in order to learn the structure of simple arithmetic word problem. Thus, for assignments with 6 sentence cards, there are _6_ *P*_3_ = 120 possible card combinations, and for assignments with 5 sentence cards, there are _5_ *P*_3_ = 60 possible card combinations.

In this study, problems posed by learners are assessed whether these are meaningful or meaningless. Meaningfulness is evaluated by how much constraints they satisfy. Table 2 shows example of meaningfulness of posed problems (learner’s answer). The first posed problem satisfied all constraints, so it must be meaningful. If a posed problem does not satisfy all the constraint but satisfy some constraint, it is also meaningful. The second and third posed problem shows the example of it. These examples show the posed problems that satisfies four constraints and only one constraint. On the other hand, if a posed problem does not satisfy any constraint, the problem is meaningless. The fourth posed problem shows an example of it. In this case the posed problem is incorrect and meaningless. To sum up, we define a meaningful problem as the problem that satisfies one or more constraints, and a meaningless problem as the problem that does not satisfy any constraint. In the analysis, we categorize posed problems and examine which kind of problem students have posed.

**Table 2:**
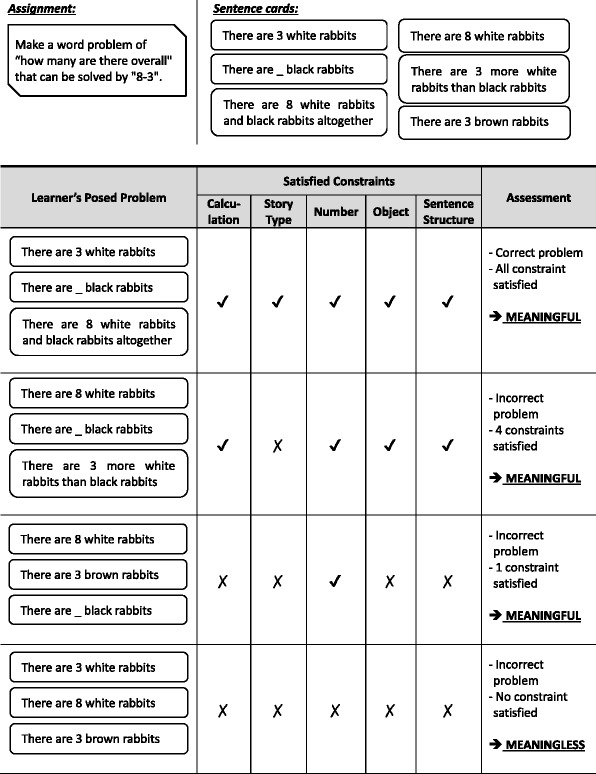
Example of some posed problems, illustrating correct and meaningful problem, incorrect but meaningful problem, and meaningless problem

As explained in “Types of constraints based on task model of problem posing” section, according to the task model of problem posing which are derived from the principle in the Triplet Structure Model, there are five constraints to be satisfied to form a correct problem. In this analysis, we categorize posed problems in terms of the numbers of satisfied constraints, and then we examine the difference between the actual number of satisfied constraints in students’ posed problems and the possible number of satisfied constraints in the assignment settings using chi-square test. Table 3 shows the proportion of actual and possible number of satisfied constraints and the result of chi-square test.

**Table 3:** Correlation analysis between actual and possible satisfied constraints in first attempt of posing problem in level 5 assignments

Asg		Number of satisfied constraints (Actual: % / Possible: %)												Actual vs possible	
		0	*p*	1	*p*	2	*p*	3	*p*	4	*p*	5	*p*	Chi-Sq	*p*
1	Actual Possible	10.3 20.0		33.3 47.5		23.1 22.5				30.8 5.0	**	2.6 5.0		<0.01	**
2	Actual Possible	0.0 15.8	**	10.5 44.2	**	15.8 30.0	+			10.5 5.0		63.2 5.0	**	<0.01	**
3	Actual Possible	0.0 6.7	+	20.5 41.7	**	12.8 31.7	*			2.6 10.0		64.1 10.0	**	<0.01	**
4	Actual Possible			38.5 75.0	**	10.3 18.3				51.3 5.0	**	0.0 1.7		<0.01	**
5	Actual Possible			33.3 76.7	**	5.1 15.0				25.6 6.7	**	35.9 1.7	**	<0.01	**
6	Actual Possible			16.2 53.3	**	32.4 40.0		35.1 3.3	**	13.5 1.7	*	2.7 1.7		<0.01	**
7	Actual Possible			14.3 38.3	*	20.0 53.3	**	14.3 3.3	*	42.9 3.3	**	8.6 1.7		<0.01	**
8	Actual Possible			12.5 46.7	**	37.5 48.3				6.3 3.3		43.8 1.7	**	<0.01	**
9	Actual Possible			20.0 63.3	**	40.0 28.3		0.0 1.7		20.0 5.0	*	20.0 1.7	**	<0.01	**
10	Actual Possible	11.1 6.7		14.8 41.7	*	25.9 31.7				22.2 10.0		25.9 10.0	+	0.043	*
11	Actual Possible	0.0 11.7	+	0.0 38.3	**	26.1 40.0				43.5 5.0	**	30.4 5.0	**	<0.01	**
12	Actual Possible	4.2 20.8	+	16.7 37.5	*	29.2 31.7				25.0 5.0	**	25.0 5.0	**	<0.01	**

**Significant difference (*p* < 0.01); *significant difference (*p* < 0.05); ^+^marginal difference (*p* < 0.1)

First, we investigate the number of satisfied constraints by all possible card combinations in each assignment. Here, *possible number* means the number of card combination that is possibly made by the students. This number is constructed based on the characteristic of correct cards and dummy cards provided in each assignment. The proportion is different depending on assignments. For example, in the first assignment, 20% of possible posed problems do not satisfy any constraint and about 70% satisfy only one or two constraints. On the other hand, in the third assignment, only 6.7% do not satisfy any constraint, and from 4th to 9th assignments there all possible posed problem will satisfy at least one constraint.

Next, we focus to investigate satisfied constraints by students’ first attempt of posing problem in each assignment, which is the first combination of three sentence cards that they selected to be assessed by Monsakun. Here, *actual number* means the number of card combination that is actually made by the students, that is, students’ answers. Table 3 shows how many constraints that students satisfied in each assignment. For example, in the first assignment, most of posed problems satisfied one to four constraints, and there are only few students (2.6%) that were able to pose the required problem. On the other hand, in second and third assignments, more than half of the students successfully posed the required problem at their first attempt.

We apply chi-square test to the counts of each number of satisfied constraints of actual and possible. If the actual number follows the proportion of possible number, the students’ problem posing in Monsakun is just in a random way. As the result, there are significant differences between actual and possible numbers in all the assignments (*p* < 0.05). This shows that students pose problems not in random way.

In addition, we pay attention to examining which kind of problems posed more or less than the possible proportion. As the result, the proportion of problems satisfying less than two constraints are less than the possible and the ones satisfying more than three constraints are more than the possible. This shows the trend in which students try to pose problems satisfying more than three constraints.

**Fig. 5:**
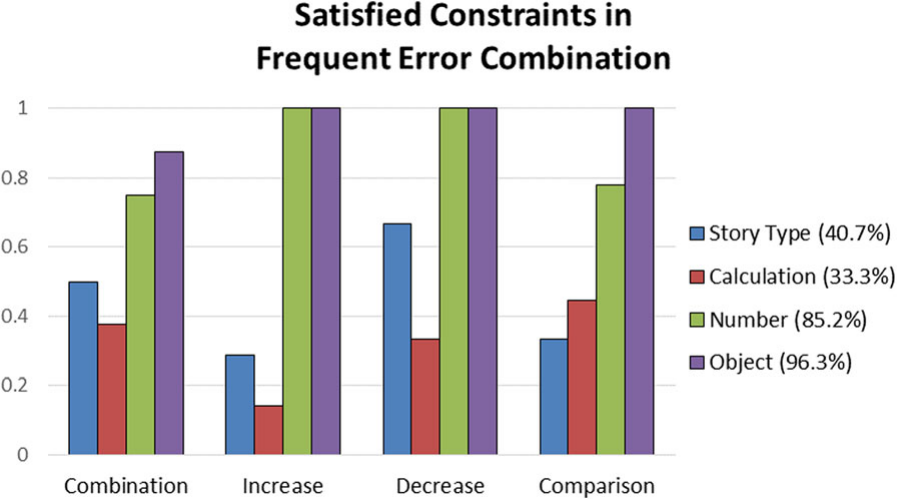
Satisfied constraints in frequent error combination

From students’ posed problems, we select frequent error combinations (>10%) and investigate the satisfied constraints in different story types. Because these combinations are incorrect answers, they automatically fulfill only four out of five constraints, whose percentages are shown in Fig. 5. The result shows that 96.3% of the frequent incorrect answers satisfy the object constraint, and 85.2% of them satisfy the number constraint. It means that the first grade of elementary school students were able to perceive the correct objects and numbers needed to pose a correct problem. However, they faced difficulties in relating the numbers with the requirement of story type and calculation, which shows lower satisfied percentage of 40.7 and 33.3%, respectively.

As explained in “Types of constraints based on task model of problem posing” section, these constraints are derived from the task model of problem posing, which is built according to the definition of arithmetic word problems in the Triplet Structure Model. From the result of analyzing the correlation between actual and possible satisfied constraints, our finding shows that most of the students were successful in understanding the given requirements in an assignment, translating them into the necessary constraints, and choosing the sentence cards that satisfies the constraints.

### Change of the trends of satisfied constraints in the same story type

In this part, we examine the trends of problem posing in the practical use of Monsakun, especially, whether learners’ trends in problem posing in Monsakun is changed during the use of Monsakun. To examine this, we studied the average of steps and mistakes in each assignment.

**Fig. 6:**
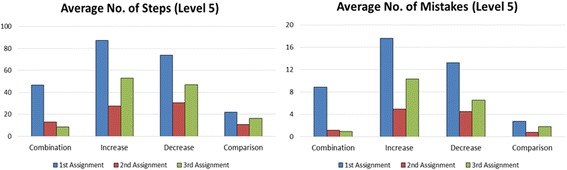
Average number of steps and mistakes in different story types in Level 5

Figure 6 shows the graph of average steps and mistakes in level 5 in different story type. The numbers shown are the total steps and mistakes from all students. There are four story types in arithmetic word problem: combination, increase, decrease, and comparison. Students are given three assignments for each story type, therefore the first to third assignments are combination problems; the fourth to sixth assignments are increase problems, and so on. We look at the average steps and mistakes in each story type by distinguishing between the first time a student pose problem in one story type and the second/third time, in consideration that a student will need to re-adjust their thinking when first time posing a different story type; thus, we assume that he would learn the problem structure in the second and third assignments in the same story type.

As shown in Fig. 6, in comparison of the first assignment in each story type, the average of steps and mistakes in the second and third assignments of the same story type are lower. This finding reflects that during the problem posing exercise using Monsakun, students might change their thinking for posing problems.

From the result shown in Table 3, in seven out of eight assignments which are second and third assignments, the proportion of actual posed problems satisfying all five constraints are significantly higher than possible numbers, in spite of no significant difference in the ones which are the first assignments where the actual numbers are very low. It can be considered that the learners learn how to think in posing problem of the same story type and they become able to pose required problems easier in the next assignments. However, it calls for further investigation of problem posing process.

## Conclusion

In this research, we have conducted analysis of posed problems in elementary school students’ problem posing activity with Monsakun in order to understand whether Monsakun encourages them to think about the structure of arithmetic word problems. The study was conducted by testing the randomness of learners' answers and analyzes the trend of them. This is a case study of analyzing part of learners’ thinking when they pose problem in a learning environment. Monsakun enables us to do such analysis because Triplet Structure Model defines the basic structure of arithmetic word problems and the constraints to form them.

This study is one step toward unveiling the work flow of students in problem posing learning environment (Birch and Beal 2008). While Hirashima et al. (2008) research showed not only students enjoyed learning problem posing with a computer-supported system but they also had better performance in math, the finding of this study shows the evidence that students were able to use the system for the intended purposes, which is to pose arithmetic word problems satisfying certain constraints. The focus of this study is on learner products of problem posing by investigating learners’ average steps and mistakes and analyzing the satisfied constraints. Even though some learners took more steps in some assignments and pose incorrect problems, they are mostly meaningful answers because they satisfy some constraints, and many learners can get to the correct answer. The correlation analysis between actual and possible posed problems shows that learners do not pose problems randomly; moreover, they are also trying to satisfy the constraints of arithmetic word problems as much as possible. We also found that learners have the difficulty to satisfy the constraints mainly about calculation and story type.

These results can be considered as an evidence of the effectiveness of Monsakun for learning arithmetic word problems of one-step addition and subtraction. From the results, it can be inferred that the learners are aware of the structure and constraints of arithmetic word problems (either completely or incompletely) and try to satisfy the constraints in posing word problems with Monsakun. This process affects learners’ understanding. The results are also important to provide support for the study of learner process in problem posing with Monsakun to determine group of learners with good or poor understanding of problem structure and to provide appropriate system assistance toward them.

A future direction of this study will be the sequential analysis of problem posing process for understanding learners’ thinking in problem posing process toward learning support. A limitation of this study is that it does not account for how learners think in problem posing, that is, the reason of learners’ choice of steps when they arranged sentence cards to make problem, made an error, and then adjusted their selection. The analytical methods used in this study focused on the result of thinking as posed problems and the overall trends of learners’ product of problem posing. The result of product analysis shows a trend that they try to satisfy problem constraints as the first step of analysis of problem posing as sentence integration. In addition to the product analysis, the process analysis of problem posing will provide much more information about learners’ thinking toward learning support. Supianto et al. (2016) have statistically analyzed the sequence of problem posing as sentence integration from the viewpoint of frequency of combinations of sentences and distance to the correct answer. This analysis has found the trap-state that can be considered as a catch for the learners in problem posing. Such statistical analysis of process with content analysis based on the constraints defined on Triplet Structure Model will contribute to analysis of learners’ thinking toward learning support.
